# Selection of endometrial carcinomas for p53 immunohistochemistry based on nuclear features

**DOI:** 10.1002/cjp2.243

**Published:** 2021-10-01

**Authors:** Eun Young Kang, Nicholas JP Wiebe, Christa Aubrey, Cheng‐Han Lee, Michael S Anglesio, Derek Tilley, Prafull Ghatage, Gregg S Nelson, Sandra Lee, Martin Köbel

**Affiliations:** ^1^ Department of Pathology and Laboratory Medicine University of Calgary Calgary AB Canada; ^2^ Department of Oncology, Division of Gynecologic Oncology University of Calgary Calgary AB Canada; ^3^ Department of Pathology and Laboratory Medicine University of Alberta Edmonton AB Canada; ^4^ Department of Pathology and Laboratory Medicine University of British Columbia Vancouver BC Canada; ^5^ Cancer Control, Alberta Health Services Holy Cross Center Calgary AB Canada

**Keywords:** endometrial cancer, molecular classification, p53, TP53

## Abstract

The World Health Organization endorses molecular subclassification of endometrial endometrioid carcinomas (EECs). Our objectives were to test the sensitivity of tumor morphology in capturing p53 abnormal (p53abn) cases and to model the impact of p53abn on changes to ESGO/ESTRO/ESP (European Society of Gynaecological Oncology/European Society for Radiotherapy and Oncology/European Society of Pathology) risk stratification. A total of 292 consecutive endometrial carcinoma resections received at Foothills Medical Centre, Calgary, Canada (2019–2021) were retrieved and assigned to ESGO risk groups with and without p53 status. Three pathologists reviewed the representative H&E‐stained slides, predicted the p53 status, and indicated whether p53 immunohistochemistry (IHC) would be ordered. Population‐based survival for endometrial carcinomas diagnosed during 2008–2016 in Alberta was obtained from the Alberta Cancer Registry. The cohort consisted mostly of grade 1/2 endometrioid carcinomas (EEC1/2; *N* = 218, 74.6%). One hundred and fifty‐two EEC1/2 (52.1% overall) were stage IA and 147 (50.3%) were low risk by ESGO. The overall prevalence of p53abn and subclonal p53 was 14.5 and 8.3%, respectively. The average sensitivity of predicting p53abn among observers was 83.6%. Observers requested p53 IHC for 39.4% with 98.5% sensitivity to detect p53abn (99.6% negative predictive value). Nuclear features including smudged chromatin, pleomorphism, atypical mitoses, and tumor giant cells accurately predicted p53abn. In 7/292 (2.4%), p53abn upgraded ESGO risk groups (2 to intermediate risk, 5 to high risk). EEC1/2/stage IA patients had an excellent disease‐specific 5‐year survival of 98.5%. Pathologists can select cases for p53 testing with high sensitivity and low risk of false negativity. Molecular characterization of endometrial carcinomas has great potential to refine ESGO risk classification for a small subset but offers little value for approximately half of endometrial carcinomas, namely, EEC1/2/stage IA cases.

## Introduction

The discovery of good prognostic *POLE* mutations in endometrial endometrioid carcinomas (EECs) and the improvement of the surrogate immunohistochemical assay for poor prognostic p53 have great potential to refine the risk stratification of patients with endometrial carcinomas [1, 2, 3, 4]. There is, however, considerable controversy on how this can be achieved. While some promote reflex testing for the molecular surrogates (*POLE*, mismatch repair [MMR] status, and p53), others argue for testing of selected cases [5, 6].

Endometrial carcinomas generally have a favorable prognosis with a 5‐year relative survival rate of 81% compared to tubo‐ovarian high‐grade serous carcinomas with approximately 40% [7, 8]. Roughly half of all patients diagnosed with endometrial carcinomas are cured with surgery alone [9]. In contrast, high risk histotypes such as endometrial serous carcinoma (ESC), endometrial clear cell carcinoma (ECCC), and carcinosarcoma (CS) are associated with an aggressive disease course [10, 11]. A newly molecularly defined aggressive histotype derived from endometrioid carcinomas, SWI/SNF‐deficient dedifferentiated/undifferentiated endometrial carcinomas (DDEC), shows the most aggressive course among all endometrial carcinomas [12]. The group with the most potential for molecular stratification is the grade 3 EEC (EEC3); several groups have shown that *POLE* mutations and p53 status further stratify this group with respect to survival [13, 14, 15, 16].

Additionally, the recent European Society of Gynaecological Oncology (ESGO), European Society for Radiotherapy and Oncology (ESTRO), and European Society of Pathology (ESP) guidelines recommend integration of certain molecular information into the risk assessment [17]. While molecular classification is encouraged in all endometrial carcinomas, the authors acknowledge that this may not be cost‐effective or feasible for all laboratories [17]. However, this also begs the question of whether cases can be selected for p53 status testing without missing abnormal cases that are at risk of progression.

We hypothesized that a substantial proportion of endometrial carcinomas (low grade and low stage) identified based on morphological features do not require additional molecular testing. Our aim was to assess the sensitivity of morphological review to select for cases that require assessment of p53 status. A secondary aim was to model the impact of abnormal p53 (p53abn) status on the ESGO/ESTRO/ESP molecular classification.

## Materials and methods

### Study cohort

We identified and retrieved 306 consecutive endometrial carcinoma hysterectomy specimens from the pathology archives at the Foothills Medical Centre/Tom Baker Cancer Centre in Calgary, Alberta, Canada, received between November 2019 and February 2021. Gynecologic oncology surgery is centralized at the Foothills Medical Centre/Tom Baker Cancer Centre and serves southern Alberta with an overall population of approximately 2 million people. Fourteen cases were excluded due to post‐neoadjuvant chemotherapy status (*N* = 3) or minimal (*N* = 8) or no residual tumor (*N* = 3) in the specimen, resulting in a final cohort of 292 cases. Original histotype and grade diagnoses were used, 94% of which were made by a group of seven pathologists with a subspecialty interest in gynecological pathology. Ethics approval was obtained from the Health Research Ethics Board of Alberta (HREBA.CC‐20‐0400).

### p53 immunohistochemistry

p53 immunohistochemistry (IHC) status was clinically reported for 29.5% of cases. For cases not clinically reported, we performed the same p53 IHC on whole formalin‐fixed and paraffin‐embedded (FFPE) tissue sections of 4 μm thickness at the Department of Pathology and Laboratory Medicine, University of Calgary, Alberta, Canada, using a previously validated protocol [4]. After 30 min of heat‐induced pretreatment using the high pH retrieval buffer, the DAKO Omnis protocol H30‐10M‐30 with the ready‐to‐use clone DO‐7 (catalog # GA61661‐2; Agilent Technologies, Santa Clara, CA, USA) was utilized. p53 was interpreted as abnormal/mutation‐type when one of the three following patterns were observed: overexpression, complete absence, or cytoplasmic; and as normal/wild‐type according to the recommended criteria [3]. Subclonal p53 was defined as the combination of normal with one or more abnormal patterns. The minimal threshold for subclonality was a cluster of at least 12 contiguous cells staining abnormally [18]. The number of subclonally abnormal patterns and their estimated extent as a percentage of the tumor were recorded.

### *POLE* sequencing

Because we hypothesized that finding subclonality on IHC in the context of MMR proficiency may indicate a *POLE* mutation, selected cases were subjected to *POLE* sequencing. Twelve cases fulfilled the following criteria: ESGO intermediate or high‐intermediate risk group, endometrioid histotype, and subclonal p53 in the context of MMR proficiency or subclonal MMR loss. DNA was extracted from FFPE tumor tissue sections or punches using the QIAamp FFPE DNA Extraction Kit (QIAGEN, Hilden, Germany). A previously described set of three redundant primers covering common hot‐spot mutations in exons 9,13, and 14 were used for Sanger sequencing in a tailed‐amplicon sequencing strategy [19]. Only mutations previously classified as pathogenic [20] were called *POLE* mutated (*POLE*mut).

### Expert review

One representative hematoxylin and eosin (H&E)‐stained slide per case was reviewed by three faculty gynecologic pathologists blinded to case characteristics. Without prior instructions or training, they were asked to categorize cases into the following five groups: 1, p53 normal/wild‐type – no IHC needed; 2, p53 abnormal/mutation‐type – no IHC needed; 3, order IHC, favor normal/wild‐type p53, rule out abnormal; 4, order IHC, possible subclonal p53; or 5, order IHC, to confirm p53 abnormal/mutant. In addition, they were asked to record the histotype (and grade if applicable) with the option to defer histotyping to after ancillary testing if the tumor showed ambiguous morphology.

### Nuclear features review

A subset of study cases (*N* = 70) was selected for a detailed review of nuclear features, focusing only on EEC or ESC cases representing different patterns of p53 staining (22 p53abn, 10 p53 subclonal, 19 p53 normal/wild‐type but with IHC ordered, and 19 p53 normal/wild‐type but without IHC ordered by study pathologists). One reviewer blinded to the p53 status recorded the following features: tumor giant cells (absent, focal, conspicuous), pleomorphism (monomorphic, pleomorphic), predominant chromatin pattern (fine, open/pale, vesicular, coarse, hyperchromatic), smudged chromatin (absent, focal, conspicuous), atypical mitoses (absent, focal, conspicuous), and nucleoli (inconspicuous, prominent, cherry red, macronucleoli, macronucleoli with inclusions). The mitotic count per 10 high‐power fields was assessed in a hot‐spot area. Counts were performed using a Nikon Eclipse Ci‐L microscope (×10 eye piece, ×40 objective; Nikon Instruments, Melville, NY, USA) with a field diameter of 0.53 mm and a field area of 0.221 mm^2^.

### Molecular risk groups based on ESGO/ESTRO/ESP

Basic clinicopathological data were abstracted, including age, % of myometrial invasion, stage, lymph‐vascular invasion (absent, focal, substantial), squamous differentiation, and clinically performed MMR protein testing. Five prognostic risk groups were defined according to the recent 2020 ESGO/ESTRO/ESP guidelines [17]: low risk (EEC1/2/stage IA), intermediate risk (EEC1/2/stage IB, EEC3/stage IA, non‐endometrioid without myometrial invasion), high‐intermediate risk (substantial lymph‐vascular invasion, EEC3, stage IB, stage II), high risk (stage III–IVA, non‐endometrioid with myometrial invasion), and advanced (residual disease, stage IVB). Dedifferentiated carcinomas were included in the non‐endometrioid group, although they are derived from endometrioid carcinomas [21].

### The Alberta Cancer Registry

For outcome analyses, an independent cohort of 4,546 endometrial carcinomas diagnosed in the province of Alberta, Canada, from 2008 to 2016 was extracted from the Alberta Cancer Registry. Follow‐up status was updated up to 21 April 2021. Endometrial cancer‐specific death was defined as death from endometrial cancer as coded by the Alberta Cancer Registry. Deaths due to non‐cancer causes or other cancers were censored for endometrial cancer‐specific death but included in the overall survival. Grade was available for 1751/3518 (49.8%) EECs.

### Statistical analyses

Pearson's chi‐squared test and analysis of variance were used for categorical and continuous data, respectively. The paired interobserver reproducibility was calculated using Cohen's kappa, and agreement was also expressed in percentages and reported as averages from the three pairs. Nominal logistic regression modeling was used to determine the predictive value of nuclear features considering their interactions. Kaplan–Meier and Cox proportional hazards survival analyses were performed. JMP14.0 software (SAS Institute, Cary, NC, USA) was used for all statistical analyses.

## Results

### Study cohort

We assembled 292 consecutive cases of endometrial carcinoma specimens from November 2019 to February 2021. The cohort consisted of 178 EEC, grade 1 (EEC1, 61.0%), 40 EEC, grade 2 (EEC2, 13.7%), 26 EEC, grade 3 (EEC3, 8.9%), 15 ESC (5.1%), 12 CS (4.1%), 9 ECCC (3.1%), 9 DDEC (3.1%; 8/9 were SWI/SNF‐deficient, all with ARID1B/ARID1A co‐loss), and one each of mesonephric‐like adenocarcinoma, large cell neuroendocrine carcinoma, and squamous cell carcinoma.

The clinicopathological parameters by main histotypes are shown in Table 1. As promoted by the 2020 World Health Organization Classification of Tumours, EEC1 and EEC2 were combined as low‐grade EECs, grades 1 and 2 (EEC1/2). Notably, 152 EEC1/2 (52.1% of all cases; 69.7% of EEC1/2) were stage IA and accordingly 147 of EEC1/2 (50.3% of all cases) were low risk by ESGO.

**Table 1:** Clinicopathological characteristics.

		EEC1/2	EEC3	ESC	CS	ECCC)	DDEC	Total	*P* value
		*N* (%)	*N* (%)	*N* (%)	*N* (%)	*N* (%)	*N* (%)		
Mean age, years (range)		63.8 (35–90)	64.8 (45–83)	71.7 (61–84)	70.8 (62–83)	71.2 (64–84)	68.4 (62–74)	64.9 (35–90)	0.011
Mean myometrial invasion, % (standard deviation)		28.64 (31.6)	41.48 (32.4)	32.2 (39.4)	53.22 (40.3)	19.5 (34.4)	72.88 (34.9)	32.28 (33.8)	0.001
Stage	IA	152 (69.7)	12 (46.2)	9 (60.0)	3 (25.0)	4 (44.4)	2 (22.2)	182 (63.0)	0.009
	IB	38 (17.4)	9 (34.6)	3 (20.0)	3 (25.0)	0 (0)	2 (22.2)	55 (19.0)	
	II	5 (2.3)	0 (0)	0 (0)	1 (8.3)	3 (33.3)	0 (0)	9 (3.1)	
	IIIA	6 (2.8)	1 (3.9)	1 (6.7)	1 (8.3)	0 (0)	0 (0)	9 (3.1)	
	IIIC1	12 (5.5)	2 (7.7)	0 (0)	4 (33.3)	1 (11.1)	3 (33.3)	22 (7.6)	
	IIIC2	4 (1.8)	1 (3.9)	1 (6.7)	0 (0)	0 (0)	1 (11.1)	7 (2.4)	
	IVB	1 (0.5)	1 (3.9)	1 (6.7)	0 (0)	1 (11.1)	1 (11.1)	5 (1.7)	
Lymph‐vascular invasion	Absent	162 (74.3)	11 (42.3)	11 (73.3)	2 (16.7)	9 (100)	0 (0)	195 (67.5)	<0.0001
	Focal	30 (13.8)	7 (26.9)	1 (6.7)	3 (25.0)	0 (0)	4 (44.4)	45 (15.6)	
	Substantial	25 (11.5)	8 (30.8)	2 (13.3)	7 (58.3)	0 (0)	5 (55.6)	47 (16.3)	
	Unknown	1	0	1	0	0	0	2	
Squamous differentiation	Absent	165 (75.7)	20 (76.9)	15 (100)	11 (91.7)	9 (100)	8 (88.9)	228 (78.9)	0.04
	Present	53 (24.3)	6 (23.1)	0 (0)	1 (8.3)	0 (0)	1 (11.1)	61 (21.1)	
MMR status	Proficient	150 (72.5)	19 (73.1)	7 (100)	4 (100)	4 (100)	3 (33.3)	187 (72.2)	<0.0001
	Subclonal	7 (3.4)	0 (0)	0 (0)	0 (0)	0 (0)	0 (0)	7 (2.7)	
	Deficient	50 (24.2)	7 (26.9)	0 (0)	0 (0)	0 (0)	6 (66.7)	63 (24.3)	
	Unknown	11	0	8	8	5	0	32	
ESGO/ESTRO/ESP risk groups	LR	147 (67.4)	0 (0)	0 (0)	0 (0)	0 (0)	0 (0)	147 (50.3)	<0.0001
	IR	30 (13.8)	10 (38.5)	4 (26.7)	2 (16.7)	3 (33.3)	0 (0)	49 (16.8)	
	HIR	18 (8.3)	11 (42.3)	0 (0)	0 (0)	0 (0)	0 (0)	29 (9.9)	
	HR	22 (10.1)	4 (15.4)	10 (66.7)	10 (83.3)	5 (55.6)	8 (88.9)	62 (21.2)	
	Advanced metastatic	1 (0.5)	1 (3.8)	1 (6.7)	0 (0)	1 (11.1)	1 (11.1)	5 (1.7)	
Total		218 (100)	26 (100)	15 (100)	12 (100)	9 (100)	9 (100)	289 (100)	

MMR status was assessed in 259/292 (88.7%) cases from which 70/259 (27.0%) showed MMR deficiency (MMRd). This included 7/259 (2.7%) cases with subclonal MLH1 or PMS2 loss. Seven cases had loss of both MSH2 and MSH6 while six cases demonstrated loss of only MSH6. Hence, at least 13/259 (5.0%) cases were possible Lynch syndrome. The distribution of MMRd by histotype is shown in Table 1. Twelve cases based on IHC subclonality findings of p53 or MMR within the intermediate and high‐intermediate risk groups were assessed for *POLE* mutation status and pathogenic *POLE* mutations (P286R, P436R, M444K; variant allelic frequency > 30%) were detected in 3/12 (25.0%) cases.

### p53 status

p53 status assessed by surrogate IHC was normal/wild‐type in 225/292 (77.1%), subclonal in 24/292 (8.2%), and abnormal/mutation‐type in 43/292 (14.7%) cases. Among p53abn cases, the following patterns were observed: 34/43 (79.1%) overexpression, 8/43 (18.6%) complete absence, and 1/43 (2.3%) cytoplasmic. The distribution of p53 status by histotype is shown in Table 2. Notably, all 15 ESC and 12 CS were p53abn. p53abn was more common in endometrioid carcinomas with solid architecture (26.9%, 7/26) compared to only 2/218 (0.9%) EEC1/2. The relationship of p53 with MMR and *POLE* status is shown in supplementary material, Table S1.

**Table 2:** p53 status by histotype.

		EEC1/2	EEC3	ESC	CS	ECCC	DDEC	Total	*P* value
		*N* (%)	*N* (%)	*N* (%)	*N* (%)	*N* (%)	*N* (%)		
p53 status	Normal/wild‐type	198 (90.8)	14 (53.9)	0 (0)	0 (0)	5 (55.6)	6 (66.7)	223 (77.1)	<0.0001
	Subclonal	18 (8.3)	5 (19.2)	0 (0)	0 (0)	0 (0)	1 (11.1)	24 (8.3)	
	Abnormal/mutation‐type	2 (0.9)	7 (26.9)	15 (100)	12 (100)	4 (44.4)	2 (22.2)	42 (14.5)	
Total		218 (100)	26 (100)	15 (100)	12 (100)	9 (100)	9 (100)	289 (100)	

**Figure 1:**
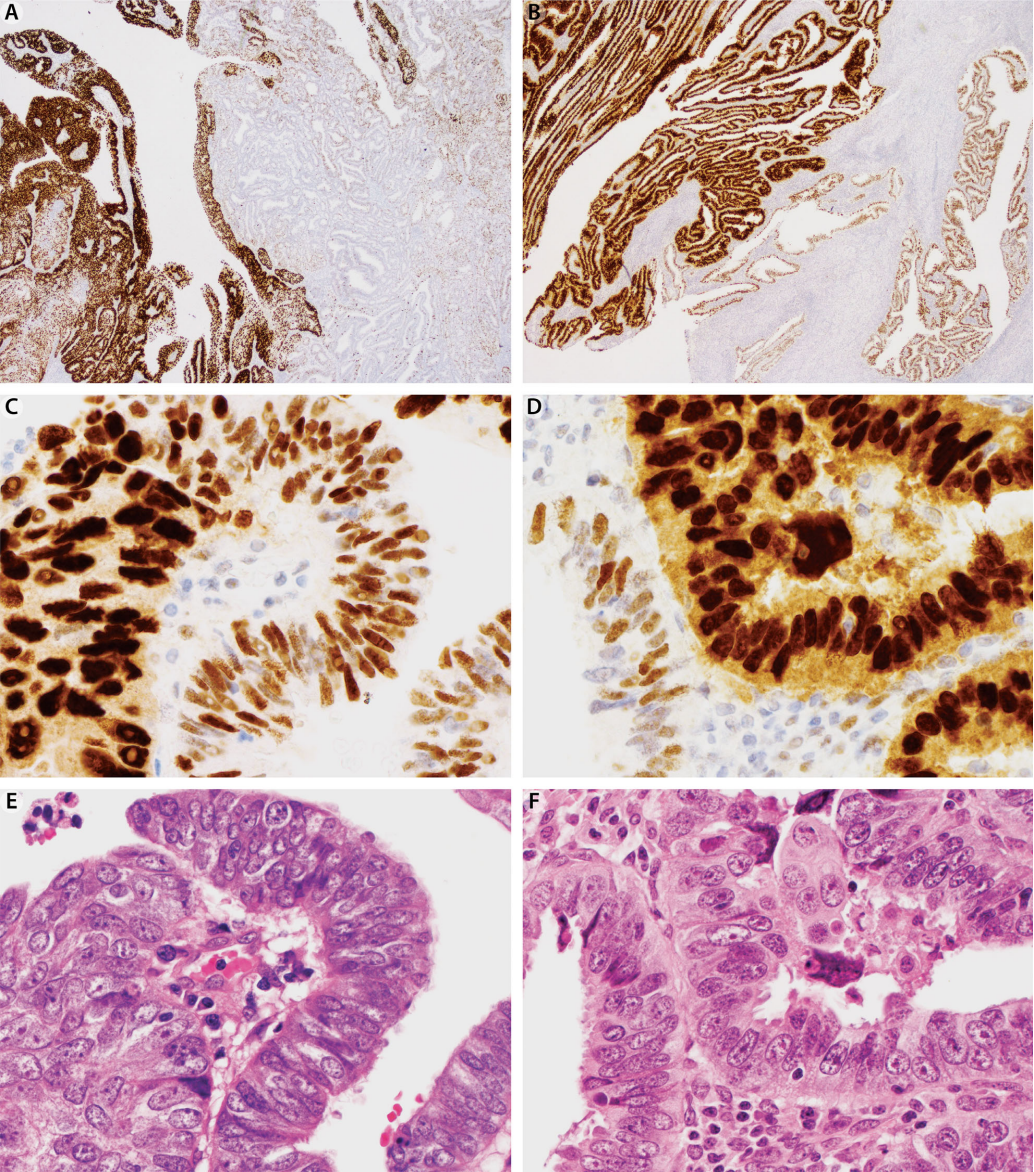
Cases showing subclonal p53 staining patterns. (A, B) Low‐power images of p53 IHC demonstrating geographic distribution of mutation‐type overexpression and normal wild‐type staining patterns. (C, D) High‐power views of p53 IHC illustrating the transition between abnormal and normal p53. (E, F) High‐power images of an H&E‐stained slide demonstrating no obvious differences in nuclear features between areas with abnormal versus normal p53 patterns.

Subclonal p53 patterns were exclusively seen in endometrioid carcinomas (EEC1/2, EEC3, DDEC; Figure 1 and Table 2). The average area of tumor demonstrating one or more abnormal subclonal patterns was 27% (range 1–95%). In 12/24 cases, subclonality was focal (<10%). Four out of 24 (16.7%) cases with subclonal patterns showed more than one abnormal pattern in combination with the normal wild‐type pattern, and 3/3 tested and therefore informative cases harbored a *POLE* mutation. Fourteen of 19 (73.7%) informative cases with subclonal p53 were either MMRd or *POLE*mut (supplementary material, Table S2).

### Prediction of p53 status by H&E morphology

We then asked three observers to predict the p53 status based on H&E morphology and indicate whether they would order p53 IHC. We considered H&E morphology as a screening test and focused on the sensitivity, i.e. probability of true positive, to predict subclonality first. The average sensitivity among the three observers to predict subclonal p53 was only 33.4% (supplementary material, Table S3). As it was so unlikely to predict subclonal p53 on H&E morphology, we focused our analysis on predicting p53abn occurring as a truncal event and grouped subclonal with normal/wild‐type. The average sensitivity to predict abnormal p53 was 83.6%, meaning there would be a false negativity rate of 16.4% (supplementary material, Table S3).

**Figure 2:**
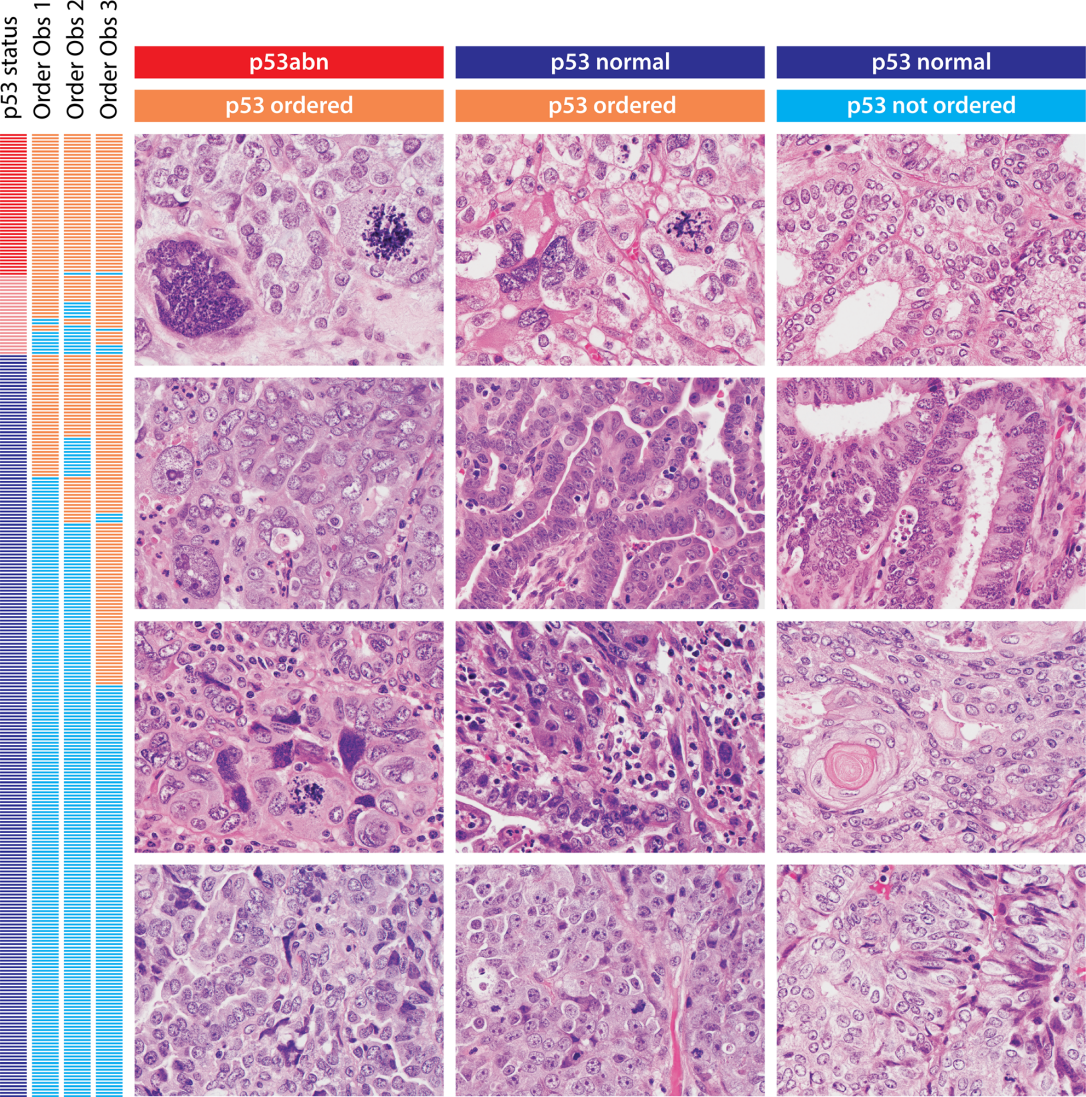
Left vertical bar shows the p53 status: p53abn (red), subclonal (pink), or normal (dark blue), followed to the right by which cases of the three observers would have ordered p53 IHC (orange) or not ordered (light blue). Note the single p53 abnormal case where IHC was not ordered. The first column illustrates p53 abnormal cases for which p53 IHC was ordered. The cases show hyperchromatic nuclei, multinucleated tumor giant cells, atypical mitoses, macronucleoli, and smudged chromatin. The middle column represents normal p53 cases for which p53 IHC was ordered, where the cases showed similar cytologic features compared to the left column. The right column shows cases with normal p53 for which p53 IHC was not ordered. The nuclear features demonstrate fine, open, or vesicular chromatin. Squamous differentiation may be seen. Degenerative smudged chromatin is a common feature, particularly at the surface of the tumor (lower right image).

We also asked the observers to select cases for which p53 status should be assessed. The three observers chose to order p53 IHC on average in 115/292 (39.4%, range 31.5–54.4%) cases (Figure 2). The average sensitivity to detect p53abn was 98.5%, and the average negative predictive value was 99.6%. Two observers missed the same p53 abnormal case (1/292, 0.3%). This particular case was diagnosed as EEC1 and is illustrated in supplementary material, Figure S1. All three observers ordered p53 for another p53abn case that was originally diagnosed as EEC2 (supplementary material, Figure S2).

### Morphological features of p53 abnormal cases

One reviewer blinded to the mutational data reviewed predefined nuclear features and mitotic activity of 70 selected cases. All features were significantly different across the p53 status but none of the individual features were perfectly sensitive in identifying p53abn (supplementary material, Table S4). However, using a nominal logistic regression model, the combination of features perfectly separated p53abn from normal cases (area under the curve of 1.000). While all features significantly contributed to the prediction, the extent of smudged chromatin, presence of pleomorphism, presence of atypical mitosis, and presence of tumor giant cells were the four most important features (Figure 2 and supplementary material, Figures S3 and S4).

In addition to the nuclear features, squamous differentiation was almost mutually exclusive to the presence of p53abn. Only two cases, one CS and one EEC3, showed both squamous differentiation and p53abn, and the EEC3 case is illustrated in supplementary material, Figure S5. However, the sensitivity of squamous differentiation appears to be limited because it only occurred in 21.1% of endometrial carcinomas (supplementary material, Table S5).

### Interobserver agreement on histotype and tumor grade

The three observers were also asked to assess histotype information. When cases selected for requiring IHC for diagnosis were excluded, which on average represented 37/292 (12.7%) cases, the average interobserver agreement for histotype when grouped into ESGO groups (low‐grade endometrioid, high‐grade endometrioid, and non‐endometrioid) was excellent with an average kappa coefficient of 0.818 (average interobserver agreement 95.3%, range 94.4–96.0%), where 91.2% (*N* = 31/34) of all disagreements were between low‐grade and high‐grade endometrioid carcinomas.

### ESGO risk groups with known molecular p53 status

**Figure 3:**
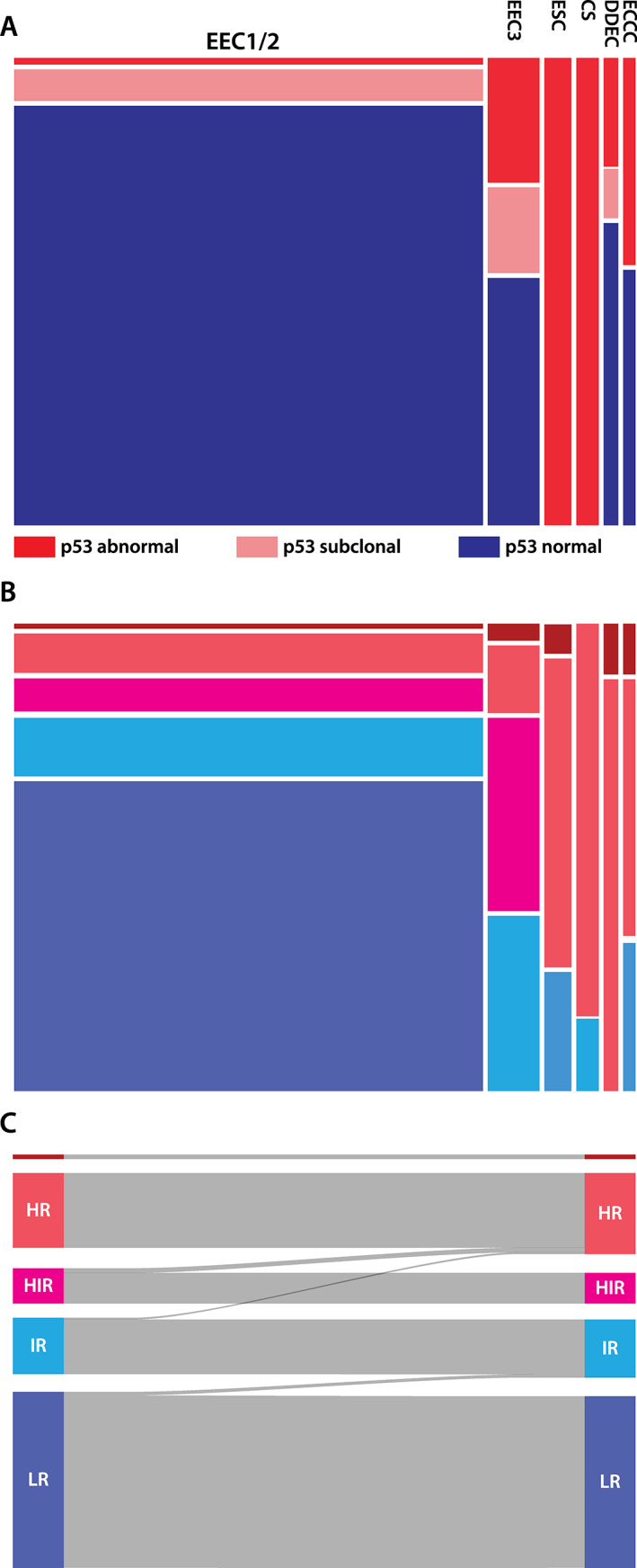
(A) Relationship between histotype and p53 status. (B) Relationship between histotype (column designation same as for A; for color coding, see C) and the ESGO risk groups. Note that approximately half of all cases are EEC1/2 and LR; a disproportionally high number of EEC3 are in the HIR group; a high proportion of non‐endometrioid and dedifferentiated carcinomas are in the HR group. (C) Sankey diagram to illustrate changes in risk groups based on p53 status: Two cases to IR and five cases to HR.

The distribution of ESGO risk groups by p53 status and histotype is shown in Figure 3. Changes due to known p53 status occurred in 7/292 (2.4%): 2 were upgraded to intermediate risk (IR) from low risk (LR), and 5 to high risk (HR) with 1 from IR and 4 from high‐intermediate risk (HIR) (Figure 3C). However, adjuvant chemotherapy would only be added for the one case that was changed from IR to HR because the other four HIR cases, which were EEC3, would have been already offered adjuvant chemotherapy.

### Model of ESGO risk groups with potential changes due to known *POLE* mutation status

*POLE* mutation status information has the potential to change the risk assignment for cases in the IR/HIR group (*N* = 78/292, 26.7%). By selecting cases from the IR/HIR group using subclonality in the absence of MMRd as the potential cause, we detected *POLE* mutations in 3/12 cases, downgrading 1 case from HIR to LR and 2 from IR to LR. However, not all IR/HIR cases were tested for *POLE*. Assuming an unbiased distribution and a *POLE* mutation prevalence of 10% in unselected endometrial carcinomas based on 12.2% reported in a large retrospective cohort, from which 82% are now considered pathogenic mutations [22, 23], we estimated that approximately 8/78 IR/HIR cases would harbor *POLE* mutations. Hence, an estimate of 8/292 (2.7%) cases could be potentially reclassified in terms of ESGO categories based on molecular information. However, as the IR group usually does not receive adjuvant therapy, we alternatively focused on the HIR group, which is more commonly offered adjuvant therapy. Within the HIR group, 15 EEC1/2 and 5 EEC3 were assigned to HIR based on substantial lymph‐vascular invasion, 6 cases were stage IB EEC3, and 3 were stage II EEC1/2. While the EEC1/2 cases would receive some form of radiation, only the 11 EEC3 in this subgroup would be considered for chemotherapy with an estimated prevalence of 1 or 2 *POLE*mut tumors.

### Population‐based survival of patients with EEC1/2

**Figure 4:**
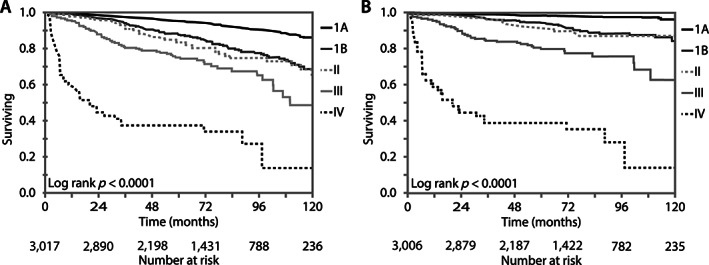
(A) Overall survival of EEC1/2* by stage. EEC1/2* stage IA shows a 5‐year overall survival of 95.3% (SE = 0.5) and a 10‐year overall survival of 86.3% (SE = 0.1). (B) Disease‐specific survival of EEC1/2* by stage. EEC1/2* stage IA demonstrates a 5‐year endometrial cancer disease‐specific survival of 98.5% (SE = 0.3) and a 10‐year endometrial cancer disease‐specific survival of 96.4% (SE = 0.8). *Grade information was not available for a subset of cases (please see main text).

The Alberta Cancer Registry recorded 4,546 endometrial carcinoma diagnoses during the period from 2008 to 2016. The annual numbers increased by 56% from 386 in 2008 to 603 in 2016, during which time there was a population growth of 17% [24]. Among all cases, 3,518/4,546 (77.4%) were diagnosed as endometrioid carcinomas (supplementary material, Figure S6). Among those, 273 were grade 3; however, grade was not available for 1,767/3,518 (50.2%) cases. Despite this limitation, we performed survival analyses for 3,245 endometrial carcinomas, predominantly grade 1 or 2 but containing approximately 270/3,245 (8.3%) potential grade 3 cases. We refer to this cohort as EEC1/2*. Of 3,245 EEC1/2* cases, 2,063 (63.6%) were diagnosed at stage IA. The 5‐ and 10‐year overall survival of stage IA, EEC1/2* cases were 95.3% (SE 0.5) and 86.3% (SE 0.1), respectively (Figure 4). The endometrial cancer disease‐specific 5‐ and 10‐year survival of this group were 98.5% (SE 0.3) and 96.4% (SE 0.8), respectively, with 41 disease‐specific deaths in 2,058 cases (Figure 4).

## Discussion

Our study shows that pathologists can reliably select endometrial carcinomas for ancillary p53 testing based on nuclear features with a negligible false negativity rate. With an acceptable interobserver variability regarding the rate of ordering, slightly more than half of all endometrial carcinomas do not require p53 testing. This group largely overlaps with the ESGO/ESTRO/ESP low risk group defined by EEC1/2 and stage IA disease. In our population‐based outcome registry, we confirm the excellent 5‐year disease‐specific survival of 98.5% for this subset. This is consistent with the conventional wisdom that many endometrial cancer patients are cured by surgery alone. We believe that there is limited advantage of universal reflex testing for p53 in endometrial carcinomas when pathologists are aware of the expected nuclear features in cases with abnormal p53 and the importance of this diagnostic and prognostic marker [25].

The rate of ordering p53 varied among the observers roughly between 40 and 60%, which was slightly higher than the original clinical ordering rate of 29.5%, possibly due to increased awareness of the diagnostic and prognostic importance of p53. The differences among observers may be related to levels of experience or thresholds, e.g. a more generous ordering to detect more subclonal cases. Nevertheless, only one challenging p53abn EEC1/2 would have been ‘missed’. This prognostic oxymoron occurred only twice in our series. Of 42 p53abn cases, 40 were of congruent high‐grade endometrioid or non‐endometrioid histotype. The one ‘missed’ p53abn EEC1 occurred in a young woman, with overwhelmingly bland nuclear features and only scattered smudged chromatin. If this case had not been confined to an endometrial polyp but had shown myometrial invasion, it would pose a dilemma whether to administer chemotherapy based purely on p53abn status [26]. According to The Cancer Genome Atlas, there is a group of low copy number endometrial carcinomas that harbor *TP53* mutations as single events, in the absence of high copy number status [1]. Perhaps, copy number analysis may be warranted in these cases. The second p53abn case represents the danger of underdiagnosing a p53abn case as EEC2 despite severe nuclear atypia: on histotype study review, this case was labelled as high‐grade endometrioid carcinoma by the observers. Overall, our recent rate of p53abn EEC1/2 is much lower compared to the historical cohorts [27, 28]. This shows the impact of pathologists as rational decision‐makers when adapting p53 IHC in their diagnostic approach [29]. However, it is important to note that the histotype and grade diagnoses were made by subspecialty gynecological pathologists from an academic center in 94% of the cases. Thus, findings may not be generalizable to community pathologists.

It has been long known among pathologists that certain nuclear features raise the possibility of ESC. Herein, we expand this empiric knowledge to other p53abn tumors. A single feature is not sufficient but a combination including the extent of smudged chromatin, the presence of pleomorphism, atypical mitoses, and tumor giant cells is predictive of the presence of a *TP53* mutation. Hence, the presence of any of these features should trigger ancillary p53 testing. We noted that degenerative smudged appearing chromatin often found on the surface of tumors was a very common feature in p53 normal tumors, which could be mistaken for the smudged chromatin associated with p53abn. Other features such as at least coarse chromatin pattern, presence of at least cherry red or macronucleoli or a high mitotic count also significantly contributed and should be a consideration in the decision‐making. While the above‐mentioned nuclear features indicate p53abn, abnormal p53 status is also associated with certain histotypes such as ESC or CS, which almost always harbor *TP53* mutations. Notably, the original clinical diagnoses of these cases were made without p53 IHC in a substantial proportion of cases based on the prototypical morphology and p53abn was only confirmed in the research setting. Beyond nuclear features and within the endometrioid histotype, the presence of squamous differentiation (although only occurring in 21.1%) can sway the decision away from p53 testing. The frequency of p53abn is significantly higher in cases with solid grade 3 architecture compared to glandular architecture; hence, the threshold for ordering should be adapted to the architectural context [30]. Future studies may evaluate whether image analysis and deep learning algorithms might be able to perform this task. Of note, a subset of p53 normal cases showed nuclear features similar to p53abn. Therefore, the presence of these nuclear features does not guarantee p53abn status and might be caused by high copy number alterations in p53 normal cases. Future studies are required to show the relationship of nuclear features to copy number and p53 status.

The observers were not able to predict subclonal p53 patterns based on morphology, and we did not observe remarkable nuclear differences between areas of p53abn versus normal p53 staining patterns when subclonal cases were reviewed for illustration. We previously reported the prevalence of subclonal p53 in endometrial carcinomas [4]. The prevalence we report herein is slightly higher (8.2%) than before (5.1%). In the current study, we used a very sensitive threshold according to our previous study to identify p53 subclonality [18]. We did this for two reasons: p53 subclonality, even if very focal, in the diagnostic setting supports a diagnosis of endometrioid carcinoma, and second, we hypothesized that subclonality could predict *POLE* mutation status. While p53 subclonality was significantly enriched for MMRd or *POLE*mut cases, it did not achieve sufficient sensitivity to select for *POLE*mut. Interestingly, the presence of more than one subclonal mutation‐type pattern seemed to be very specific for *POLE*mut based on 3/3 informative cases [29]. León‐Castillo *et al*. reported that the majority of the 3% of ‘multiple classifier’ cases defined by co‐occurring p53 abnormality with MMRd or *POLE*mut showed subclonal p53 [4, 31]. We also detected that 1.5% of cases would be ‘multiple classifier’ cases based on subclonal p53. However, we did not detect a single ‘multiple classifier’ case based on p53abn as truncal or clonal alteration affecting all tumor cells. Of note, our assessment was largely restricted to MMRd/p53abn cases because of the selected *POLE*mut testing. In our previous study, we detected 3/177 (1.7%) MMRd/p53abn cases and 1/177 (0.6%) *POLE*mut/p53abn [4]. Therefore, we conclude that the co‐occurrence of *POLE*mut with truncal p53abn is uncommon. However, this also highlights the need to distinguish subclonal p53 from truncal p53abn particularly with respect to the upper cut‐off: two cases in our series showed subclonal p53 with 95% mutation‐type pattern and only 5% normal wild‐type pattern. These cases easily enter the differential when considering a diagnosis of ESC. Subclonal p53 status is not relevant for prognosis if these cases are MMRd or *POLE*mut [32]. However, future studies should attempt to clarify the prognostic and predictive significance of the small group of subclonal p53 cases without MMRd or *POLE*mut. If this proves to be clinically significant, the inability to detect this subset of cases would be a significant limitation of morphological selection for p53 testing. It goes without saying that subclonality is best assessed in well‐fixed samples such as whole sections or representative endometrial biopsies while tissue microarrays may sample only one or the other clone. Sequencing may not be able to accurately infer subclonality because this depends on the ratio of accurate tumor cellularity estimates versus allelic frequency. Future studies may evaluate subclonal cases microdissected for the p53abn and normal components to confirm a double‐hit alteration in *TP53* in the p53abn component, preferentially including the copy number alteration status.

p53 status changes ESGO risk group assignment in a small number of patients (7/292, 2.4%), which is similar to another recent study (17/594, 2.9%) [28]. Of note, in our study, only one additional patient would have been offered adjuvant chemotherapy. Although the number is small, it is important to note that chemotherapy has been shown to be effective in p53abn tumors [31]. An important difference between the recent similar study by Imboden *et al*. and our study is the recommendation regarding p53 testing. Imboden *et al*. recommend testing all stage I and II tumors (largely EEC1/2), requiring 72.0% of cases to be tested [28]. In contrast, we propose a morphology/nuclear feature driven approach in which roughly half of the EEC1/2 cases with bland nuclear features would not require p53 IHC testing.

A limitation in our study due to limited funding is that the *POLE* status was only known in a few cases. Nevertheless, we model that the presence of *POLE* mutation could change ESGO risk categories in about 3% (8/292) of cases. Our assumption regarding *POLE*mut was based on prevalence estimates of 10% in unselected patients and assuming an unbiased distribution [22, 23]. We also assumed that only cases from the IR/HIR group would change risk categories. However, Imboden *et al*. reported that 12/22 *POLE*mut cases were found within the HR group. The HR group in our study accounted for 21.6% of the cohort, from which 64.5% (40/62) were stage III/IV and 35.5% (22/62) were non‐endometrioid carcinomas (stage I/II) with myometrial invasion. Only the latter would change risk groups based on *POLE*mut status. Selecting HIR/IR groups and HR stage I/II non‐endometrioid carcinomas would require *POLE*mut testing in 100/292 (34.2%) cases. Based on our experience that prototypical non‐endometrioid carcinomas do not harbor *POLE* mutations [13, 33], testing of non‐endometrioid carcinomas in the IR (9 cases) and HR (22 cases) groups could be further restricted to cases with ambiguous/mixed histology, *POLE* phenotype [34], and cases with multiple subclonal p53 patterns. Taken together, in our study, about 5% (15/292, 8 *POLE*mut + 7 p53abn) potentially change ESGO risk categories based on molecular information, which is slightly lower than the 7% reported by Imboden *et al*. [28]. Yet, when it comes to changes in adding or withdrawing chemotherapy, only 2–3 patients (1–2 *POLE*mut + 1 p53abn) would be affected.

It is reassuring that histotypes aligned well with the p53 status but also that there was high interobserver agreement in histotype diagnoses: >95% agreement among the observers from a single institution after excluding ~12% ambiguous cases that would require ancillary work‐up. This is much higher compared to recent studies selecting for difficult high‐grade cases and shows the importance of studying interobserver agreement in an unselected series [35, 36]. The main disagreement occurred between EEC1/2 and EEC3, highlighting the importance of second opinions and consideration of p53 testing in borderline cases. Notably, in this contemporary cohort, not a single original diagnosis of mixed carcinoma was made [37]. It is our clinical practice to subject cases with ambiguous or ‘mixed’ morphology to MMR testing and occasionally *POLE* mutation testing, which may render an integrated diagnosis of MMRd or *POLE*‐mutated endometrioid carcinoma. This avoids the prognostically antagonistic ‘*POLE*‐mutated serous carcinoma’ [25]. The relatively high number of dedifferentiated carcinomas, almost all defined by co‐loss of ARID1B/ARID1A, highlights the importance of ARID1B assessment (in our case by IHC), which in itself represents a higher risk diagnosis than high‐grade endometrioid carcinoma in more cases than those upgraded by p53abn [12, 21].

Our study shows that molecular classification of endometrial carcinoma has great potential to refine the classification of patients into ESGO risk groups for a small subset of patients. p53 IHC is an important tool that has enhanced the robustness of integrative histotyping and molecular classification. Selection of cases for ordering IHC can be based on nuclear features with consideration of solid architecture and absence of squamous differentiation. Our data support the ESGO/ESTRO/ESP recommendation that ‘in low‐risk endometrioid carcinomas, the molecular classification may not be required’ [17]. If molecular testing resources are limited, ancillary p53 testing can be targeted to a morphologically defined subset of endometrial carcinomas.

## Author contributions statement

EYK contributed to cohort assembly, histopathological evaluation, interpretation of results, and drafting of the manuscript. NJPW and SL contributed to histopathological evaluation and data collection. CA, MSA, PG, GSN and DT were involved in data collection and interpretation. CL was involved in interpretation of results and drafting of the manuscript. MK contributed to study conception, design, and planning; histopathological evaluation; interpretation of results; and drafting of the manuscript. All authors were involved in writing the paper and had final approval of the submitted and published versions.

## Supporting information


**Figure S1.** p53 abnormal endometrial endometrioid carcinoma, grade 1, missed by two observers
**Figure S2.** Endometrial endometrioid carcinoma, grade 2 (EEC2), based on severe nuclear atypia, with abnormal p53
**Figure S3.** Expanded illustrations of histologic features
**Figure S4.** Nominal logistic regression model of nuclear features predictive of abnormal p53 status
**Figure S5.** An endometrial endometrioid carcinoma, grade 3, with both squamous differentiation and abnormal p53
**Figure S6.** Endometrial carcinoma cases diagnosed in Alberta, Canada, between 2008 and 2016 by histotype distribution and by year from the Alberta Cancer Registry

**Table S1.** p53 across MMR and *POLE* status
**Table S2.** Subclonal p53 and MMR cases
**Table S3.** Diagnostic test table for p53 prediction and ordering
**Table S4.** Nuclear feature review table
**Table S5.** Squamous differentiation across p53 status
