# Supplementary material for: Selection of endometrial carcinomas for p53 immunohistochemistry based on nuclear features
Source: J Pathol Clin Res. 2021 Oct 1;8(1):19–32. doi: 10.1002/cjp2.243 (PMC8682942; doi:10.1002/cjp2.243)
Supplement: Supplementary file 1 — Figure S1. p53 abnormal endometrial endometrioid carcinoma, grade 1, missed by two observers Figure S2. Endometrial endometrioid carcinoma, grade 2 (EEC2), based on severe nuclear atypia, with abnormal p53 Figure S3. Expanded illustrations of histologic features Figure S4. Nominal logistic regression model of nuclear features predictive of abnormal p53 status Figure S5. An endometrial endometrioid carcinoma, grade 3, with both squamous differentiation and abnormal p53 Figure S6. Endometrial carcinoma cases diagnosed in Alberta, Canada, between 2008 and 2016 by histotype distribution and by year from the Alberta Cancer Registry [file CJP2-8-19-s002.pdf]

Selection of endometrial carcinomas for p53 immunohistochemistry based on nuclear features

EY Kang *et al.* *J Pathol Clin Res* DOI: 10.1002/cjp2.243

Supplementary Figures

Figure S1. p53 abnormal endometrial endometrioid carcinoma, grade 1 missed by 2 observers. Representative images from the hematoxylin and eosin-stained slide show focal smudged chromatin. This case was a stage IA case confined to an endometrial polyp with no myometrial invasion. The patient did not receive adjuvant therapy and had no evidence for recurrent disease after 14 months.

Figure S2. Endometrial endometrioid carcinoma, grade 2 (EEC2), based on severe nuclear atypia, with abnormal p53. p53 immunohistochemistry (IHC) was ordered by all observers. Representative images of the hematoxylin & eosin slide show quite diffuse nuclear atypia, cherry-red nucleoli (right upper image), and smudged chromatin (left lower image). Abnormal overexpression p53 IHC is shown in the right lower image. This case was not diagnosed as EEC2 by all observers; in some instances, this was diagnosed as EEC grade 3 or as needing IHC for diagnosis. Since this case belonged to the low-risk group according to European Society of Gynaecological Oncology (ESGO) guidelines (stage IA, no myometrial invasion, no lymph-vascular invasion), abnormal p53 would have changed this case to intermediate-risk by ESGO guidelines, which still would not warrant adjuvant therapy. An integrated diagnosis of p53-abnormal EEC grade 3 would also have changed the case only to intermediate-risk.

Figure S3. Expanded illustrations of histologic features seen in cases that had A) abnormal p53 status where observers requested immunohistochemistry, B) p53 normal status where observers indicated they would order immunohistochemistry, and C) p53 normal status where observers did not ask for immunohistochemistry.

Figure S4. Nominal logistic regression model of nuclear features predictive of abnormal p53 status.

Figure S5. An endometrial endometrioid carcinoma, grade 3, with both squamous differentiation and abnormal p53.

Figure S6. Endometrial carcinoma cases diagnosed in Alberta, Canada between 2008 and 2016 by histotype distribution and by year from the Alberta Cancer Registry.

Figure S1

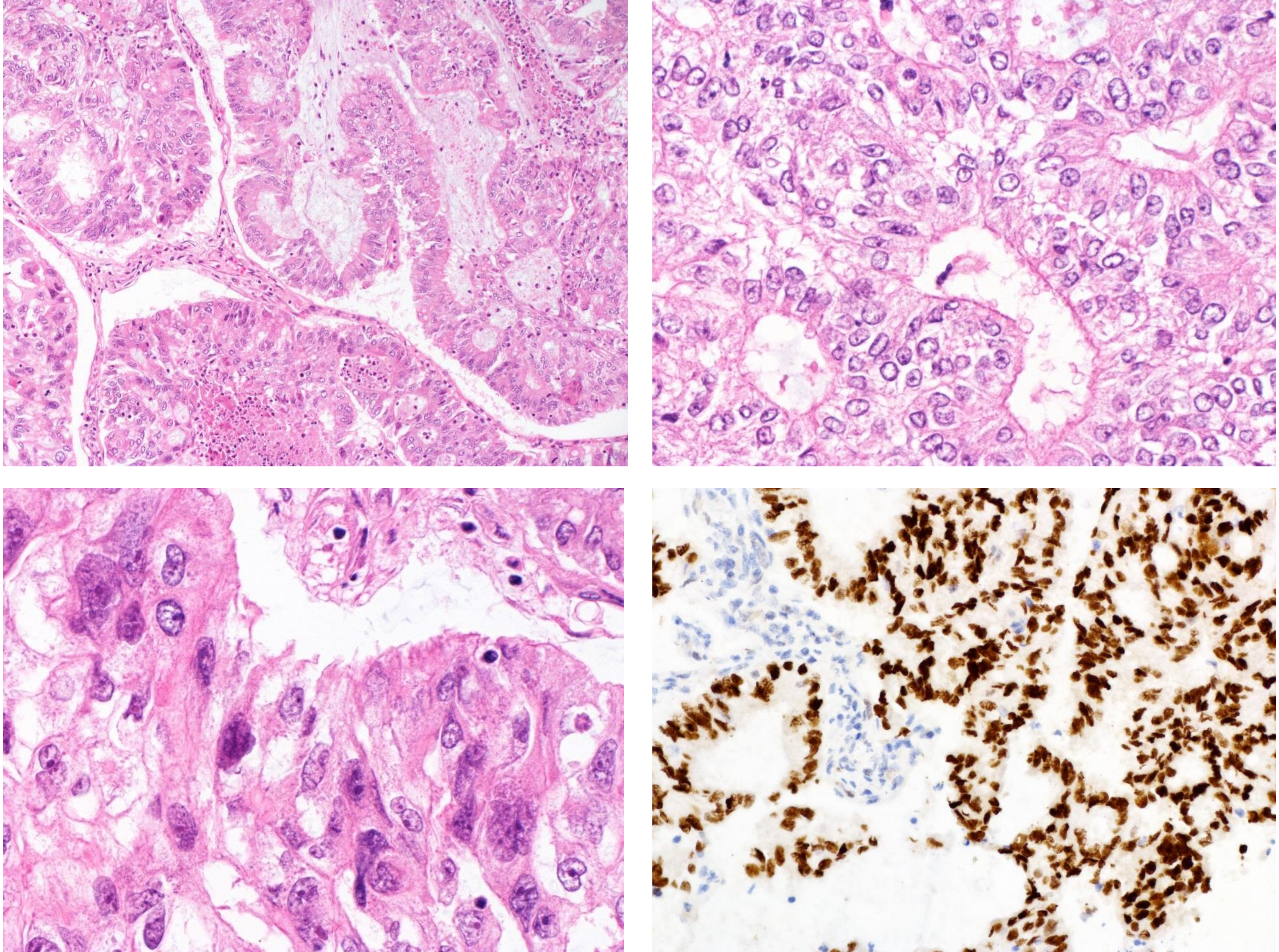

Figure S2

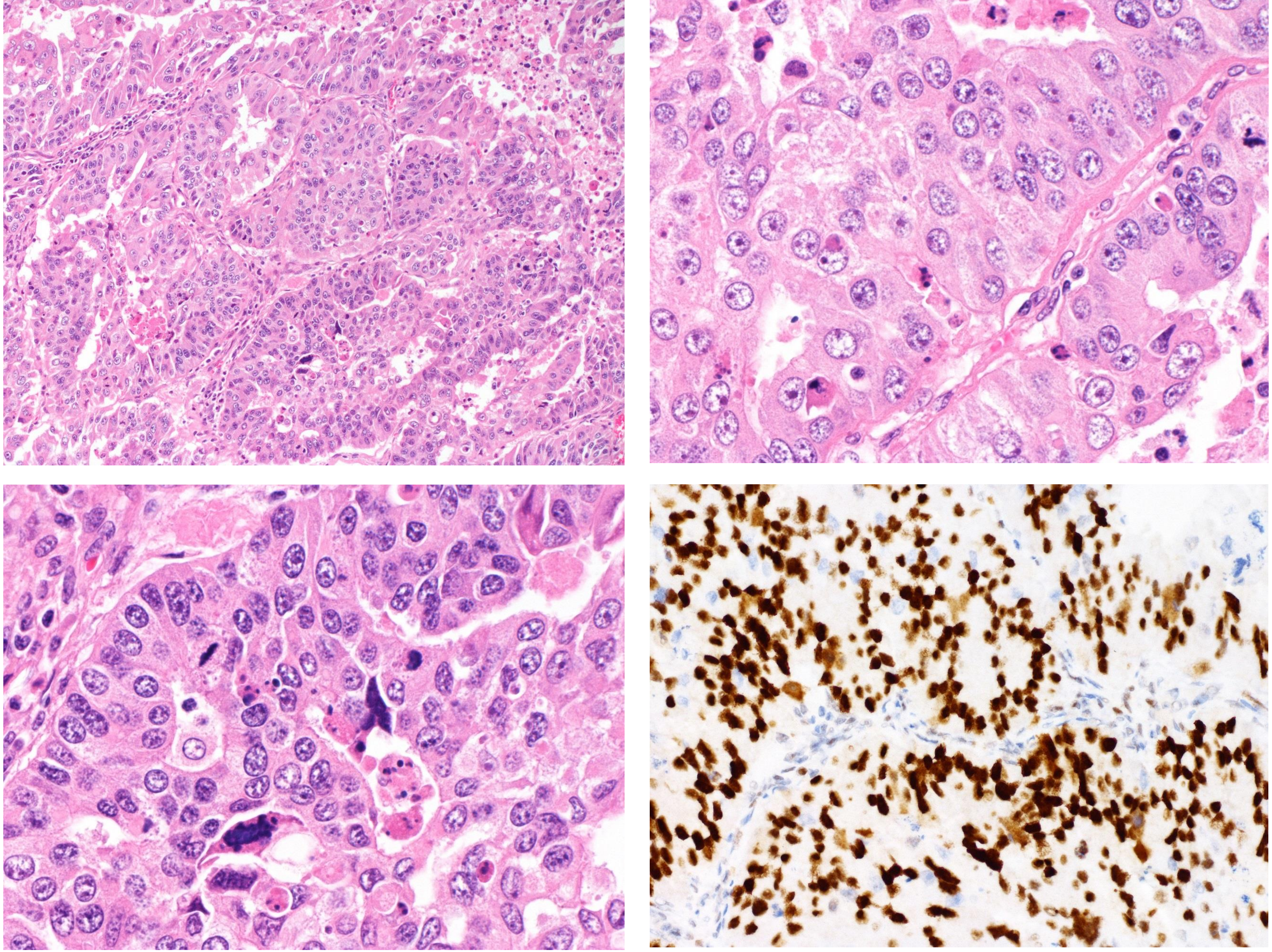

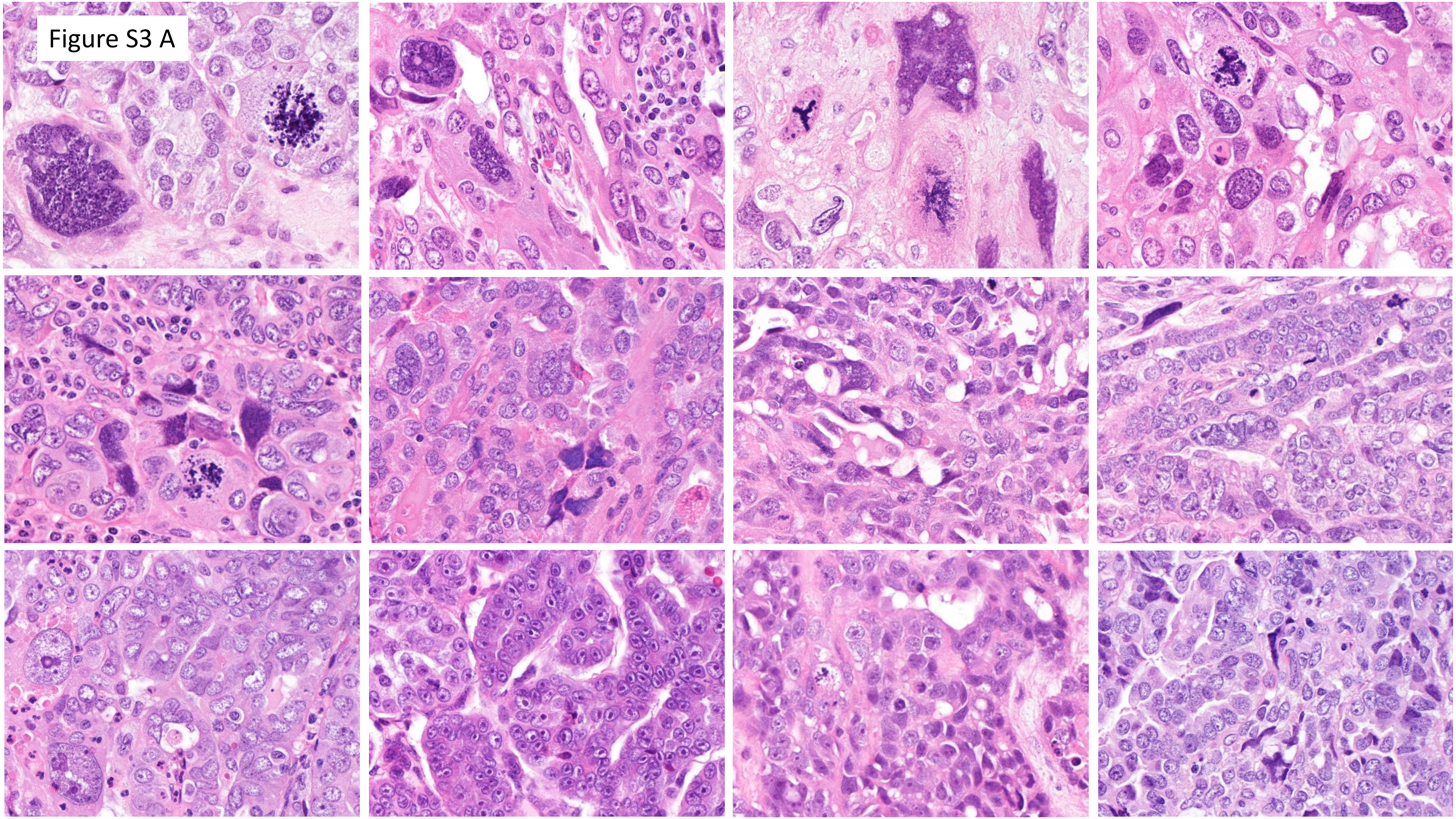
Figure S3 A

Figure S3 B

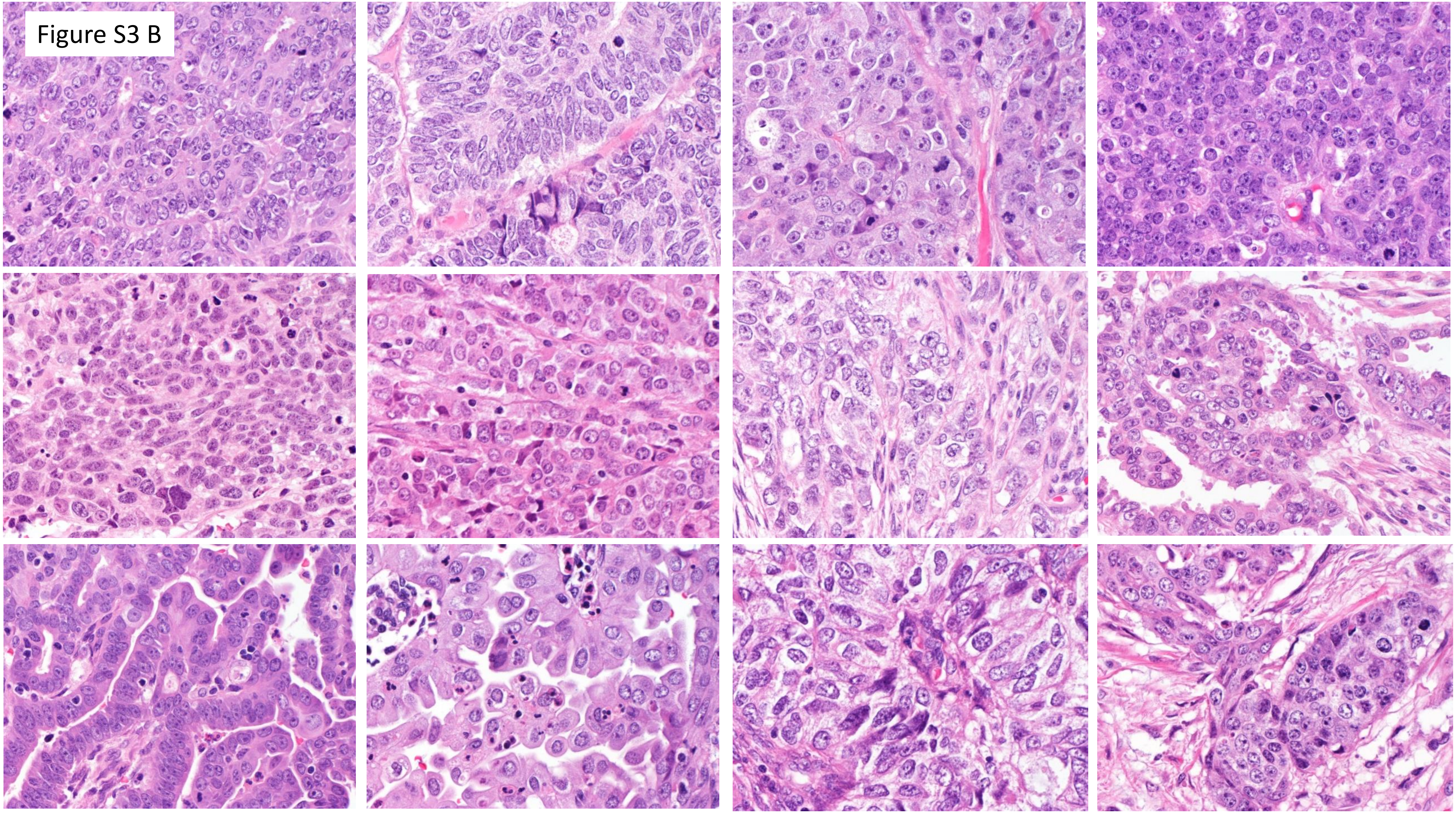

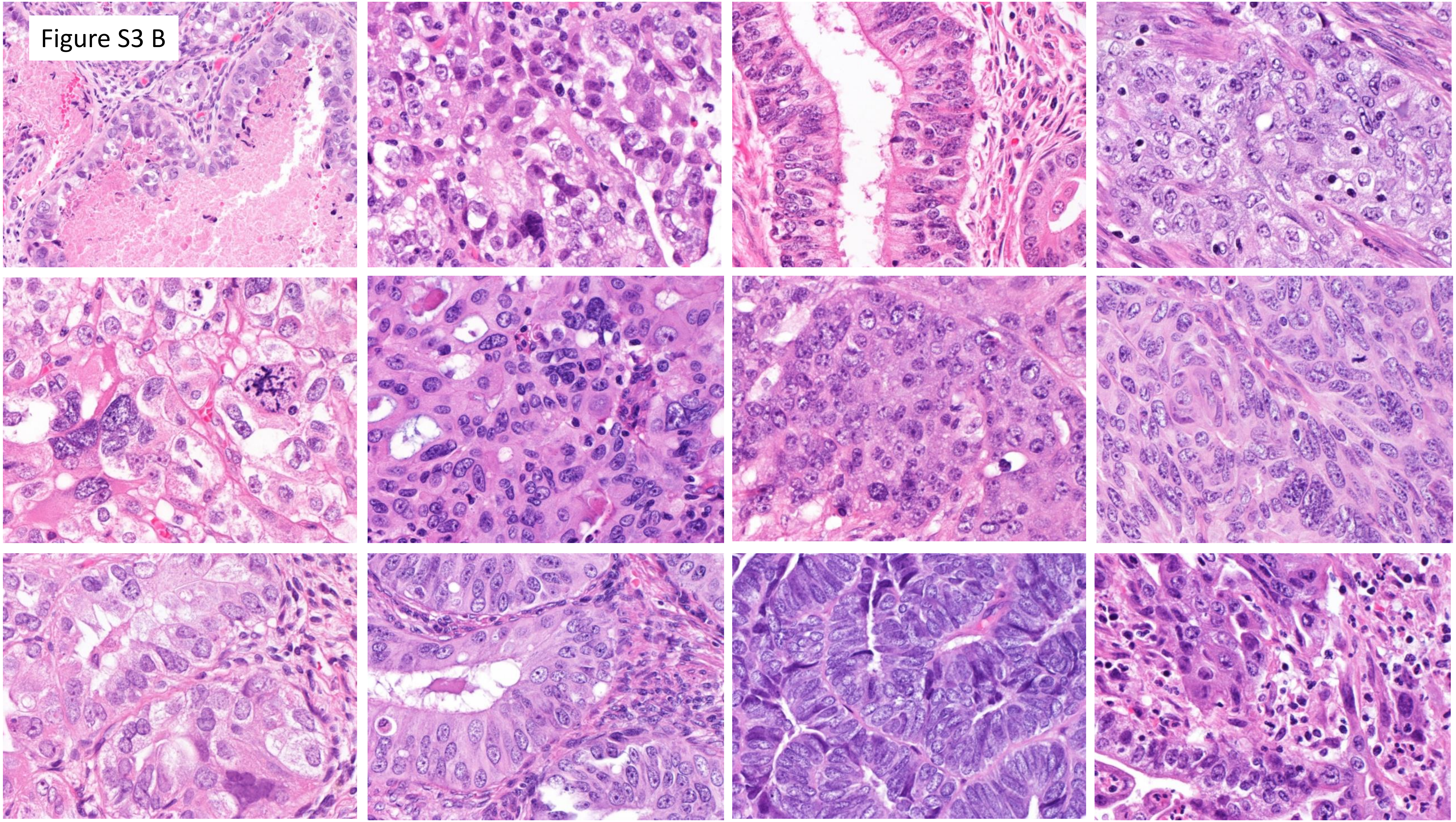
Figure S3 B

Figure S3 C

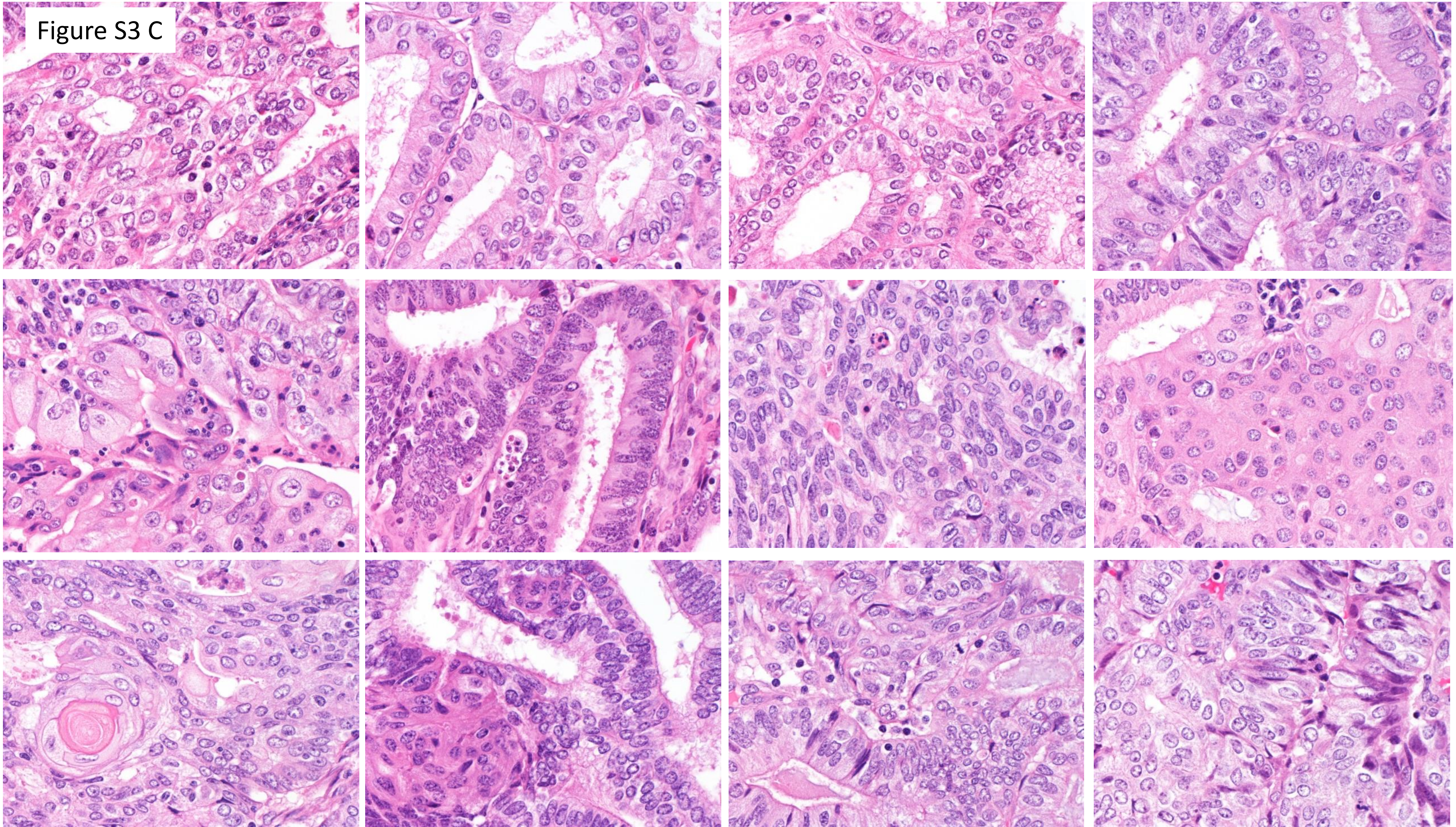

Figure S4

Nominal Logistic Fit for p53 excl SC

Effect Likelihood Ratio Tests

Source	Nparm	DF	L-R ChiSquare	Prob>ChiSq
Tumor giant cells	2	2	92.6222479	<.0001*
Pleomorphism	1	1	410.171025	<.0001*
Predominant chromatin pattern 3	3	3	17.8870259	0.0005*
Smudged chromatin	2	2	503.633645	<.0001*
Atypical mitoses	2	2	272.380089	<.0001*
Nucleoli	3	3	8.86173001	0.0312*
Mitotic count (/10HPF)	1	1	7.63835155	0.0057*

Using p53 excl SC='Abn' to be the positive level

AUC
1.00000

Confusion Matrix

Training

Actual	Predicted Count	
p53 excl SC	Abn	WT
Abn	22	0
WT	0	37

Figure S5

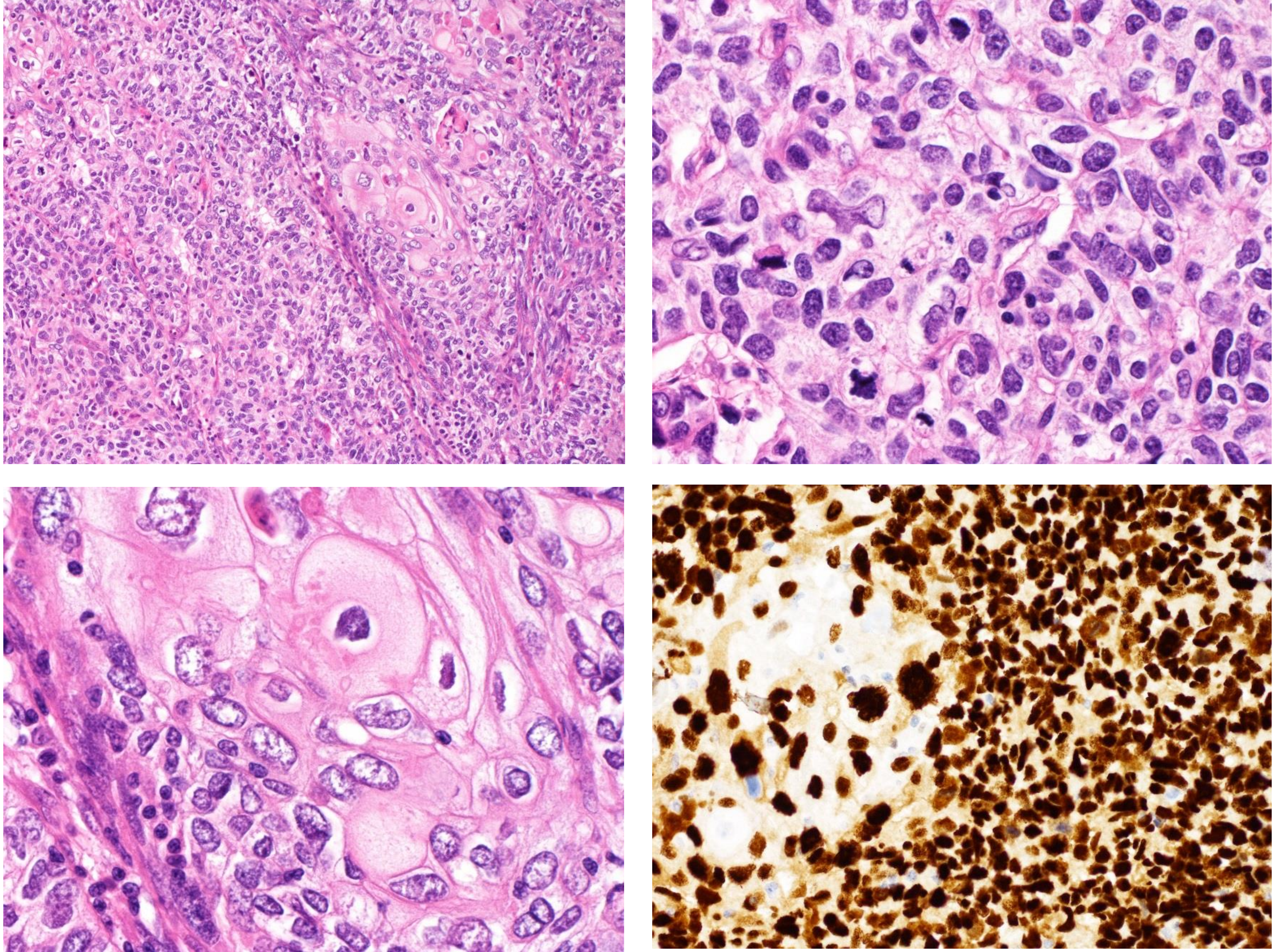

Figure S6

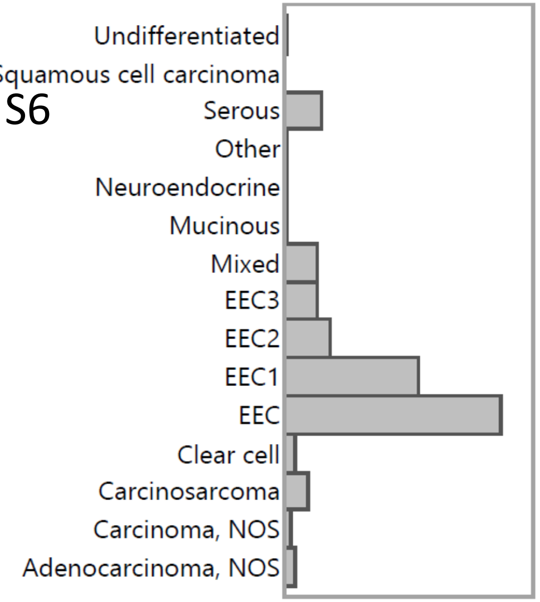

Frequencies

Level	Count	Prob
Adenocarcinoma, NOS	83	0.01826
Carcinoma, NOS	49	0.01078
Carcinosarcoma	193	0.04245
Clear cell	81	0.01782
EEC	1767	0.38869
EEC1	1102	0.24241
EEC2	376	0.08271
EEC3	273	0.06005
Mixed	260	0.05719
Mucinous	15	0.00330
Neuroendocrine	10	0.00220
Other	15	0.00330
Serous	301	0.06621
Squamous cell carcinoma	2	0.00044
Undifferentiated	19	0.00418
Total	4546	1.00000

N Missing 0
15 Levels

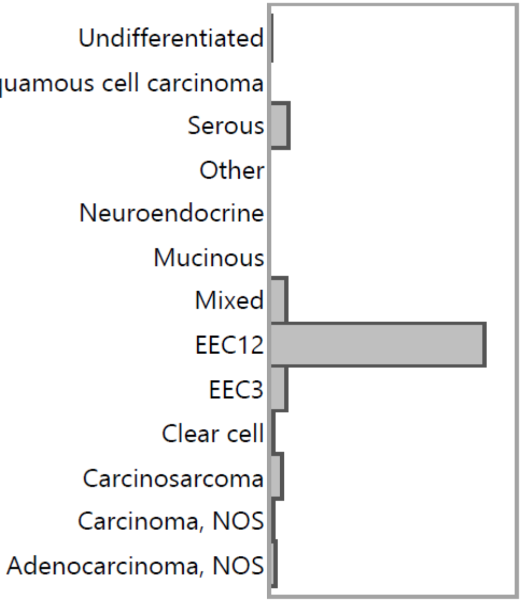

Frequencies

Level	Count	Prob
Adenocarcinoma, NOS	83	0.01826
Carcinoma, NOS	49	0.01078
Carcinosarcoma	193	0.04245
Clear cell	81	0.01782
EEC3	273	0.06005
EEC12	3245	0.71381
Mixed	260	0.05719
Mucinous	15	0.00330
Neuroendocrine	10	0.00220
Other	15	0.00330
Serous	301	0.06621
Squamous cell carcinoma	2	0.00044
Undifferentiated	19	0.00418
Total	4546	1.00000

N Missing 0
13 Levels

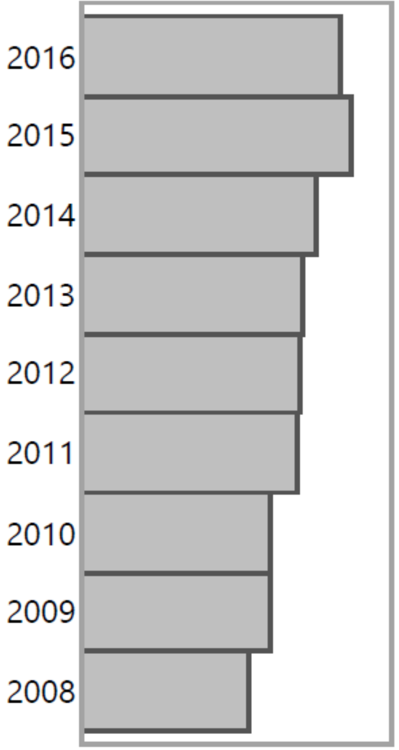

Frequencies

Level	Count	Prob
2008	386	0.08491
2009	436	0.09591
2010	435	0.09569
2011	498	0.10955
2012	508	0.11175
2013	514	0.11307
2014	541	0.11901
2015	625	0.13748
2016	603	0.13264
Total	4546	1.00000

N Missing 0
